# Immunological Pathways in Sarcoidosis and Autoimmune Rheumatic Disorders—Similarities and Differences in an Italian Prospective Real-Life Preliminary Study

**DOI:** 10.3390/biomedicines11061532

**Published:** 2023-05-25

**Authors:** Miriana d’Alessandro, Laura Bergantini, Sara Gangi, Edoardo Conticini, Dalila Cavallaro, Paolo Cameli, Fabrizio Mezzasalma, Luca Cantarini, Bruno Frediani, Elena Bargagli

**Affiliations:** 1 Respiratory Diseases Unit, Department of Medical and Surgical Sciences & Neurosciences, University of Siena, 53100 Siena, Italy; 2 Rheumatology Unit, Department of Medicine, Surgery & Neurosciences, University of Siena, 53100 Siena, Italy; 3 Diagnostic and Interventional Bronchoscopy Unit, Cardio-Thoracic and Vascular Department, Azienda Ospedaliera Universitaria Senese (AOUS), University Hospital of Siena, 53100 Siena, Italy

**Keywords:** sarcoidosis, autoimmune rheumatic disorders, adaptive immune system

## Abstract

Background: The pathogenesis of sarcoidosis involves T cells and B lymphocytes that produce autoantibodies. We compared the expression of different T and B cell subsets in sarcoidosis and three B-mediated rheumatic diseases that can affect the lungs in an attempt to identify similarities and differences that distinguish these diseases. Methods: The study included patients referred to Siena University Hospital’s respiratory disease and rheumatology units. Patients were enrolled prospectively and consecutively. Healthy volunteers were also included. Multicolor flow cytometry was performed on phenotype T and B cell subsets. Multivariate analysis was carried out to reduce the dimensionality of the data. Results: Fifteen patients had a diagnosis of sarcoidosis, fourteen idiopathic inflammatory myopathies (IIM), five granulomatosis with polyangiitis (GPA), ten microscopic polyangiitis (MPA), and seven were controls. Thirty-five T and B cell subsets were phenotyped, 15 of which were significantly different in sarcoidosis, B-mediated rheumatic disorders, and controls. Principal components analysis distinguished the four groups of patients with a total explained variance of 54.7%. A decision tree was constructed to determine which clustering variables would be most useful for distinguishing sarcoidosis, IIM, MPA, and GPA. The model showed regulatory T helper cells (Th-reg) > 5.70% in 91% of sarcoidosis patients as well as Th-reg ≤ 5.70 and Th17 > 43.27 in 100% of MPA. It also showed Th-reg ≤ 5.70, Th17 ≤ 43.27 and Tfh-reg ≥ 7.81 in 100% of GPA patients, and Th-reg ≤ 5.70, Th17 ≤ 43.27 and Tfh-reg ≤ 7.81 in 100% of IIM patients. Conclusion: The immune cell profile sheds light on similarities and differences between sarcoidosis and B-mediated rheumatic diseases. Sarcoidosis and autoimmune diseases show similar patterns of cellular immune dysregulation, suggesting a common pathogenic pathway that may provide an opportunity for further understanding autoimmunity and exploring biological therapies to treat sarcoidosis.

## 1. Introduction

Sarcoidosis is an inflammatory disorder of unknown etiology, characterized by systemic granulomatous inflammation mainly affecting the lungs [1]. Diagnosis is usually determined by multidisciplinary evaluation of clinical, immunological, and radiological findings. Bronchoalveolar lavage (BAL) and biopsy are recommended in the diagnostic workup. This minimally invasive procedure enables the identification of immune cell subtypes in the alveolar district via bronchoscopy [2]. A CD4^+^/CD8^+^ ratio greater than 3.5 is considered highly specific for sarcoidosis [3]. Sarcoidosis is attributed to a number of factors, including autoantigen-specific T cells, antibodies producing B lymphocytes, and autoimmune inflammation. The pathogenesis of sarcoidosis involves autoimmune components, despite the fact that it is not classified as an autoimmune disease [4]. Several striking parallels with autoimmune disorders and common variable immunodeficiency have been discovered in recent studies into T cell plasticity in sarcoidosis, challenging the long-held paradigm of T helper type 1 (Th1) [5].

Among autoimmune rheumatic disease (ARD), there are ANCA-associated vasculitis (AAV) and idiopathic inflammatory myopathy (IIM), both of which are B-mediated rheumatic disorders. A loss of immune tolerance leads to the activation of the immune system against self-tissues and organs. These disorders can be systemic or organ-specific. ANCA-associated vasculitis in the form of granulomatosis with polyangiitis (GPA) and microscopic polyangiitis (MPA) affects the respiratory system, kidneys, and upper and lower extremities [6]. A necrotizing vasculitis characterized by inflammation of the vascular wall in conjunction with peri- and extravascular granulomatosis (similar to sarcoidosis) is called GPA. Idiopathic inflammatory myopathy (IIM) has different forms characterized by immune-mediated inflammation of the striate muscle, CD4-mediated damage (dermatomyositis), and immune alterations [7], such as myositis-specific and myositis-associated antibodies, usually with well-defined clinical patterns that may be more aggressive when IIM is associated with rapidly progressive interstitial lung disease.

Three factors influence the pathogenesis of the abovementioned ARD: genetic predisposition (associated with major histocompatibility antigens and other immunoreactive molecules), environmental exposure to physical and chemical agents, and infection. Hormonal factors and stressful life events may also play a part in its development. Autoimmune disorders exhibit an inflammatory milieu due to leukocytosis and acute-phase proteins [8]. 

Together, these findings emphasize the need for comparative studies on sarcoidosis and B-mediated rheumatic diseases for insights into the failure of peripheral tolerance linked to the development of these diseases. However, only one article reported the expression of immune checkpoint molecules (PD1, CTLA4, TIGIT) on T- and NK-cells that negatively regulate the T-cell immune function in granulomatous diseases including sarcoidosis and AAV [9].

No data are available on adaptive immune cell profiles shared by ARD and sarcoidosis, despite emerging evidence that these conditions are related in terms of cellular immune dysregulation. Here, we used the relative distribution of different T and B cell subset percentages to find similarities and differences between sarcoidosis and three B cell-mediated rheumatic conditions that can affect the lungs. Adaptive immune profiles were also compared with those of a group of healthy volunteers.

## 2. Materials and Methods

### 2.1. Study Population

Patients referred to Siena Regional Referral Centre for sarcoidosis and to the rheumatology unit of Siena University Hospital were prospectively and consecutively enrolled in the study. The diagnosis of sarcoidosis was confirmed by multidisciplinary discussion according to American Thoracic Society/European Respiratory Society/World Association of Sarcoidosis and Other Granulomatous Disorders (ATS/ERS/WASOG) guidelines. IIM patients were diagnosed according to the 2017 EULAR criteria and/or Peter and Bohan criteria for dermatomyositis, anti-synthetase positivity, and typical clinical features of anti-synthetase syndrome, while AAV patients, including granulomatosis with polyangiitis (GPA) and microscopic polyangiitis (MPA), were diagnosed according to the 2022 ACR/EULAR classification criteria. Patients diagnosed with polymyositis were excluded because no specific diagnostic criteria are available for this disorder which is currently diagnosed by exclusion [10]. 

Peripheral blood samples were collected from patients for immune profiling. Their demographic, clinical, and radiological data were entered into a prestructured electronic database. Written informed consent to participate in the study was obtained from all patients and healthy controls who had no history of concomitant pathologies, were not on medication, and had normal lung function. The study was approved by the regional ethical review board of Siena, Italy (C.E.A.V.S.E., Comitato Etico Area Vasta Sud Est, Markerlung 17431 and RHELABUS 22271), and complied with the Declaration of Helsinki.

### 2.2. Gating Strategy 

Multicolor flow cytometric analysis was performed, and the gating strategy is reported in the Supplementary Materials. The first tube was to detect B cell subsets and included the following mAbs: CD19-PE-Cy7 (SJ25C1), CD24-APC-Cy7 (ML5), CD38-FITC (HB-7), CD5-PerCP-Cy5.5 (L17F12), CD1d-PE (all from BD Biosciences, San Jose, CA, USA), CD45-APC (REA747 Miltenyi Biotec S.r.l., 40133 Bologna, Italy), and CD27-BV510 (M-T271 BioLegend, San Diego, CA, USA). 

The second tube was to detect follicular T cells and included mAbs: CD4-APC-Vio770, CD45RA-PE-Vio770, CCR6-APC, CXCR3-VioBright FITC CXCR5 PerCP-Cy5.5, and CCR4-PE, all from Miltenyi Biotec (40133 Bologna, Italy).

The third tube was to phenotype regulatory T cells according to the BD Human Regulatory T Cell Cocktail (CD4-FITC (SK3), CD25-PECy7 (2A3), CD127 Alexa Fluor^®^ 647, BD, San Jose, CA, USA), CXCR5 PerCP-Cy5, CD8 horizon450, and CD3 APC-Cy7 (OKT3) (Biolegend, San Diego, CA, USA).

Gating was carried out using Kaluza software 2.1 (Beckman Coulter, Inc., Danvers, MA, USA): details are shown in the Supplementary Materials. The T and B cell subsets are also described in the Supplementary Materials.

### 2.3. Statistical Analysis

All values were expressed as the mean, interquartile range (IQR), or standard deviation, as appropriate. The Shapiro–Wilk test was used to check for normal distribution of values. ANOVA (Kruskal–Wallis test) and the Dunn test for multiple comparisons were performed using a non-parametric method. For categorical variables, Fisher’s exact or Chi-squared tests were used to compare proportions between groups. Spearman tests were conducted to determine correlations between clinical and immunological parameters. 

Multivariate analysis was conducted on cell subsets (immature B cells, Tfh-reg, Th effector, Th naïve, Th-reg, Th17.1, Th1, Th17naïvenaive, Tc-reg, CD8 and Tfc17) that differed in a statistically significant way between sarcoidosis, IIM, MPA, and GPA groups. A two-dimensional representation of multidimensional data was used to identify trends in immunological features by supervised principal component analysis (PCA). Four groups (sarcoidosis, IIM, MPA, and GPA) were formed to identify the best thresholds for group classification. Based on the Gini criterion, a classification and regression decision tree were constructed to identify optimal clustering variables. We evaluated the accuracy of binary classifiers by confusion matrix, creating a series of test/training partitions. A *p*-value less than 0.05 was considered statistically significant. Statistical analysis was performed with GraphPad Prism 9.4 (GraphPad, San Diego, CA, USA) and XLSTAT 2021 software (Lumivero, Denver, CO, USA).

## 3. Results

### 3.1. Study Population

Forty-four patients were enrolled consecutively and prospectively at diagnosis; none of them were being treated at the time of sampling. Fifteen patients had sarcoidosis (mean ± standard deviation age 55 ± 11.3 years): nine were women, and there was a prevalence of never-smokers (57%). All sarcoidosis patients were in Scadding radiological stage II, indicating thoracic lymph node enlargement and sarcoid involvement of the respiratory system. Fifteen patients had a diagnosis of AAV (mean ± standard deviation age 61 ± 14.8 years; 8 females, 61% never-smokers). Ten out of fifteen AAV patients had MPA, and 5 out of 15 had GPA. Fourteen patients had a diagnosis of IIM (mean ± standard deviation age 58 ± 12.5 years; 7 females, 75% never-smokers). Five out of fourteen IIM patients had dermatomyositis (3 TIF1gamma-positivity and 2 MDA5-positivity), and 9 out of 14 had anti-synthetase syndrome (6 Jo1-positivity and 3 PL7-positivity). One patient with polymyositis was excluded from the analysis. Eleven healthy volunteers (mean ± standard deviation age 58 ± 12.4 years; 6 females) were also enrolled in the study.

### 3.2. Comparison of T and B Cell Subsets in Sarcoidosis, IIM, GPA, and MPA Groups

Immature B cells were more numerous in sarcoidosis than IIM samples (*p* = 0.0035). Follicular Th regulatory cells (Tfh-reg) were less numerous in sarcoidosis and IIM than in MPA (*p* = 0.0258 and *p* = 0.0267, respectively) and GPA samples (*p* = 0.0297 and *p* = 0.0389, respectively). T effector cells were also less numerous in sarcoidosis than in MPA (*p* = 0.0259). T naïve cells were more numerous in IIM than in sarcoidosis (*p* = 0.0136). T helper regulatory cells were more numerous in sarcoidosis than in IIM (*p* = 0.0019), MPA (*p* = 0.0033), and GPA samples (*p* = 0.0029). Follicular Th 17.1 were more numerous in sarcoidosis than in IIM (*p* = 0.0450). Th1 cell percentages were higher in GPA than in IIM (*p* = 0.0227) and sarcoidosis (*p* = 0.0129). They were also higher in MPA than in IIM (*p* = 0.0127) and sarcoidosis samples (*p* = 0.0126). Th17 cell percentages were higher in GPA than in IIM (*p* = 0.0353) and sarcoidosis (*p* = 0.0435). 

T cytotoxic naïve cells were less numerous in sarcoidosis than in IIM (*p* = 0.0062), MPA (*p* = 0.0035), and GPA samples (*p* = 0.0297). T cytotoxic regulatory cells were more numerous in sarcoidosis than in IIM (*p* = 0.0456), whereas CD8-positive cell percentages were lower in sarcoidosis than in IIM samples (*p* = 0.0152). Follicular T cytotoxic-17 (Tfc17) cell percentages were higher in GPA (*p* = 0.0439) and IIM (*p* = 0.0492) than in sarcoidosis samples.

### 3.3. Comparison of T and B Cell Subsets between Patients (Sarcoidosis, IIM, GPA, and MPA) and Controls

**Figure 1:**
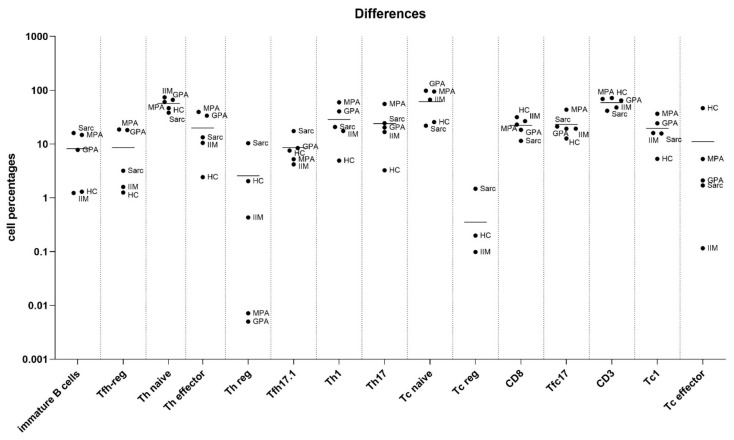
Differences in T and B cell subsets (immature B, follicular Th regulatory (Tfh-reg), Th effector, Th naïve, Th-reg, T follicular Th 17.1 (Tfh17.1), Th1, Th17, Tc naïve, Tc reg, CD8, follicular Tc 17 (Tfc17), CD3, Tc1, and Tc effector) in blood samples from sarcoidosis, idiopathic inflammatory myopathy (IIM), granulomatosis with polyangiitis (GPA), and microscopic polyangiitis (MPA) patients and healthy controls (HC). Cell percentages are expressed as mean and standard deviation; *p* values are reported in Results. Abbreviations: Sarc, sarcoidosis; Tfh-, T follicular helper; Th-, T helper; Tfc, T follicular cytotoxic; Tc-, T cytotoxic; CD-, cluster of differentiation.

**Figure 2:**
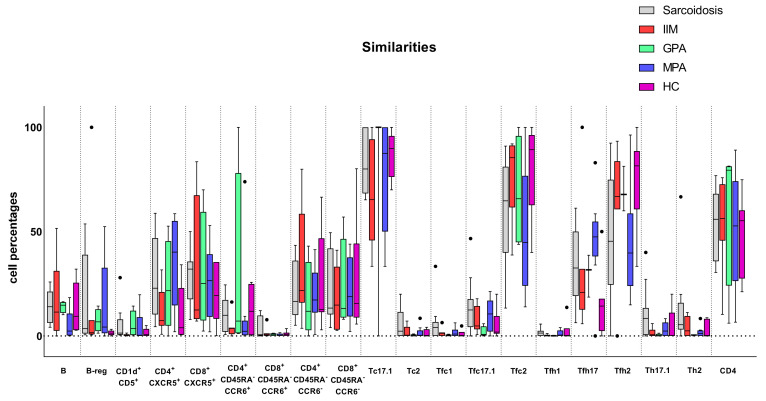
Similarities of T and B cell subsets (B cells, Breg cells, CD1d^+^CD5^+^, CD4^+^CXCR5^+^, CD8^+^CXCR5^+^, CD4^+^CD45RA^−^CCR6^+^, CD8^+^CD45RA^−^CCR6^+^, CD4^+^CD45RA^−^CCR6^−^, CD8^+^CD45RA^−^CCR6^−^, T cytotoxic 17.1 (Tc17.1), Tc2, follicular T cytotoxic 1 (Tfc1), Tfc17.1, Tfc2, Tfh1, Tfh17, Tfh2, Th17.1, Th2, and CD4) in blood samples from sarcoidosis, idiopathic inflammatory myopathy (IIM), granulomatosis with polyangiitis (GPA), and microscopic polyangiitis (MPA) patients and from healthy controls (HC). Cell percentages are expressed as mean and standard deviation. Abbreviations: Sarc, sarcoidosis; Tfh-, T follicular helper; Th-, T helper; Tfc, T follicular cytotoxic; Tc-, T cytotoxic; CD-, cluster of differentiation.

Figure 1 and Figure 2 show T and B cell subset similarities and differences in patients and controls.

Immature B cells were more numerous in controls than in sarcoidosis patients (*p* = 0.0182). CD3+ cells were less numerous in sarcoidosis than in control samples (*p* = 0.015). They were also more numerous in MPA (*p* = 0.0237) and GPA (*p* = 0.0329) than in control samples. Th effector cells were more numerous in sarcoidosis and MPA than in control samples (*p* = 0.002 and *p* = 0.0151, respectively). Tfh-reg were more numerous in MPA than in control samples (*p* = 0.0203). Th-reg cells were more numerous in sarcoidosis than in control samples (*p* = 0.0048) but less numerous in MPA than in control samples (*p* = 0.0133). Th1 cell subsets were more numerous in GPA and MPA than in control samples (*p* = 0.0363 and *p* = 0.0227, respectively). Th17 cell percentages were higher in GPA than in control samples (*p* = 0.0308). 

Tc effector cells were more numerous in sarcoidosis than in control samples (*p* = 0.038). Tc naïve cells were less numerous in the control samples than in MPA samples (*p* = 0.0151). CD8+ cells were more numerous in the control samples than in the sarcoidosis samples (*p* = 0.0302). Tc1 cell percentages were lower in controls than in MPA (*p* = 0.0474) and GPA patients (*p* = 0.0363). 

### 3.4. Multivariate Analysis

**Figure 3:**
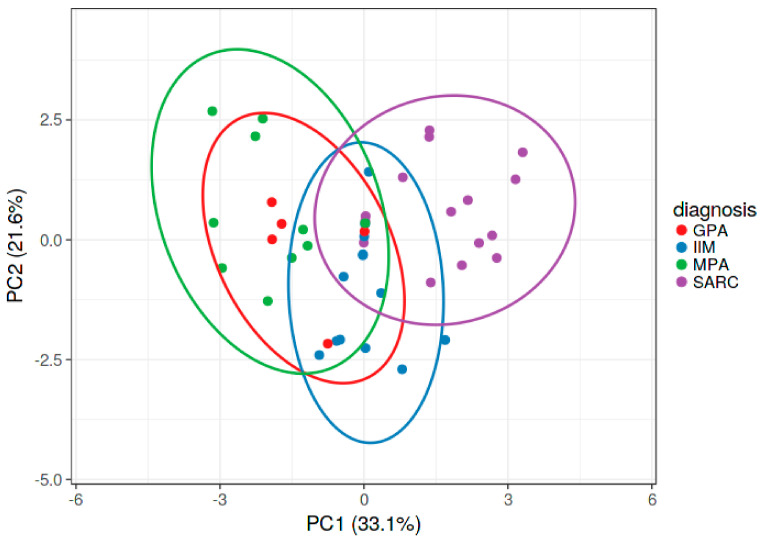
Principal component analysis (PCA) distinguished the four groups of patients (sarcoidosis, idiopathic inflammatory myopathy (IIM), granulomatosis with polyangiitis (GPA), and microscopic polyangiitis (MPA)) according to T and B cell subsets (immature B cells, follicular Th regulatory (Tfh-reg), Th effector, Th naive, Th-reg, follicular Th 17.1 (Tfh17.1), Th1, Th17, T cytotoxic naïve (Tc naïve), regulatory T cytotoxic (Tc-reg), CD8, and follicular T cytotoxic 17 (Tfc17)). The first and second components explained 60.47% of the total variance. Abbreviations: Sarc, sarcoidosis; Tfh-, T follicular helper; Th-, T helper; Tfc, T follicular cytotoxic; Tc-, T cytotoxic; CD-, cluster of differentiation.

The PCA plot (Figure 3) distinguished four disease clusters. Sarcoidosis, IIM, MPA and GPA were separated on the basis of immature B cells, Tfh-reg, Th effector, Th naive, Th-reg, Tfh17.1, Th1, Th17, Tc naïve, Tc-reg, CD8, and Tfc17. The first and second components explained 33.1% and 21.6% of the variance, respectively.

**Figure 4:**
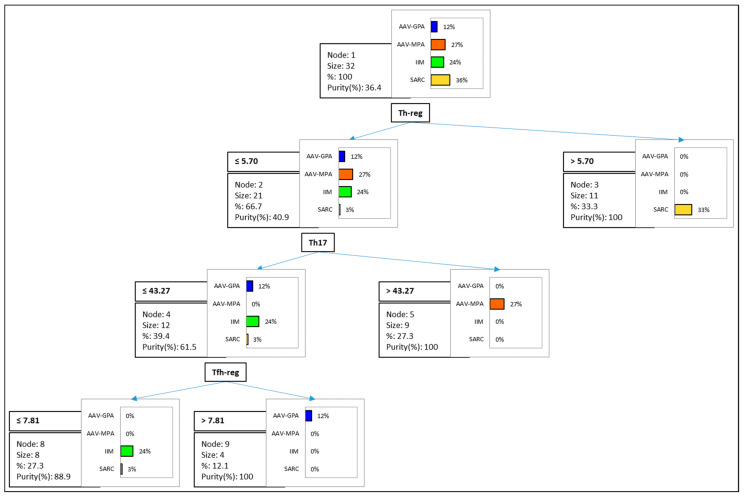
Decision tree showing the successive steps by which the model identified the variables that best divided the four groups of patients (sarcoidosis, idiopathic inflammatory myopathy (IIM), granulomatosis with polyangiitis (GPA), and microscopic polyangiitis (MPA)). The dependent variables were T and B lymphocyte subsets: immature B cells, follicular Th regulatory (Tfh-reg), Th effector, Th naïve, Th-reg, follicular Th 17.1 (Tfh17.1), Th1, Th17, T cytotoxic naïve (Tc naïve), regulatory T cytotoxic (Tc-reg), CD8, and follicular T cytotoxic 17 (Tfc17). Abbreviations: Sarc, sarcoidosis; AAV-, ANCA-associated vasculitis; Tfh-, T follicular helper; Th-, T helper; Tfc, T follicular cytotoxic; Tc-, T cytotoxic; CD-, cluster of differentiation.

An analysis of the decision-tree models (with cross-validation by confusion matrix) was conducted to identify the best clustering variables that can distinguish between the groups of sarcoidosis, IIM, MPA, and GPA (Figure 4). The model showed: Th-reg > 5.70% for 91% of sarcoidosis patients; Th-reg ≤ 5.70 and Th17 > 43.27 for 100% of MPA patients; Th-reg ≤ 5.70, Th17 ≤ 43.27, and Tfh-reg > 7.81 for 100% of GPA patients; Th-reg ≤ 5.70, Th17 ≤ 43.27, and Tfh-reg ≤ 7.81 for 100% of IIM patients.

## 4. Discussion

An important objective of the present preliminary study was to determine the differences and similarities in immune profiles between sarcoidosis patients and patients with IIM and AAV. We also compared all profiles with those of healthy controls. Multivariate analysis of the data reduced the number of dimensions, and a total variance of 54.7% was obtained based on T and B cell subsets. The total variance was determined by a classification and regression tree, showing good clustering variables for sarcoidosis, IIM, MPA, and GPA. Treg cell percentages clustered 91% of sarcoidosis patients with a cut-off of 5.7%, while 100% of IIM, MPA, and GPA patients were clustered on the basis of Treg, Th17, and Tf-reg cell percentages.

Sarcoidosis and ARD patients often have lymphopenia. We found lower CD3 and CD8 cell percentages in sarcoidosis than in IIM and control samples; T cells were also below the normal range of controls in MPA and GPA patients. 

The immune response to inflammatory diseases is generally regulated by B cells [11]. In line with this, our findings suggest that peripheral immature B cells were more abundant in pulmonary sarcoidosis and AAV than IIM in both conditions [12]. The chemokine receptor (CXCR)5 is highly expressed in Tfh cells, which are essential for these cells to migrate toward B cell follicles during the germinal centre reaction. This receptor is essential for the formation of plasma and memory B cells during said reaction. CD4+ and CD8+ T cells with a memory phenotype express CXCR5 in a small subset of peripheral lymphocytes (14% and 2%) [13]. As a result of the discovery of the circulating counterpart to GC Tfh, these cells can now be studied in clinical settings. As a result, several strategies have been employed to define Tfh in human blood, analyzing the CXCR5+ molecule within the CD4 T cells or measuring the CXCR5+ molecule in the plasma. As a first step, we examined the CXCR5^+^ population in circulating memory CD4 T cells since this strategy is often conducted prior to analyzing subsets of Tfh1, Tfh2, or Tfh17 [14].

The helper and cytotoxic subsets of T follicular cells (Tfh and Tfc) expressed the main Th17-cell chemokine receptors implicated in chronic inflammatory conditions. This population of cells is referred to as Tfh17 and Tfc17. Autoimmune diseases typically have a preponderance of Tfh17 cells [15]. Although T cells include a subset of Th17 cells coexpressing IFN-gamma (termed Th17.1 cells), abundant in sarcoid lungs [14], no information is available on the distribution of Tfh17.1 cells. Here, we found similar increased expression of Tfh17.1 cells in sarcoidosis and AAV but not in IIM patients.

Although they are transcriptionally and phenotypically distinct from other CD8 T cell subsets, Tfc cells may play a role in antibody-mediated autoimmune disease progression. There is limited information available about Tfc distribution in sarcoidosis and acute lung diseases. Recently, Zhai et al. demonstrated elevated Tfc cell percentages in patients with primary Sjogren syndrome, especially between those with lung involvement and controls [16]. 

A wide variety of effector subsets are generated by T cells. They include Th cells, T follicular cells (Tfc), and regulatory T cells (Tregs). T cells, Tfc, and Tregs stimulate target cells, whereas Th cells, Tfs, and Tregs suppress them [17]. 

As a subpopulation of T cells, Tregs control the immune system, maintain tolerance to self-antigens, and prevent the occurrence of autoimmune diseases. The role of Tregs in sarcoidosis pathogenesis has long been suspected, although research findings are inconsistent [18]. 

CD127 (IL-7Ra) is highly expressed by naive T cells, which are precursors of effector and memory T cells [19]. T cells are increased by CD127, a growth factor that restores and maintains lymphocyte counts, including CD4+ and CD8+ T cells, increasing the number of functioning T cells. Mature naive and memory T cells survive, multiply and differentiate by activating the IL-7/IL-7R pathway. Disruption of this pathway has been implicated in the pathogenesis of several ARDs, but no data are available for IIM or AAV. In line with this, we found a higher proportion of Th- and Tc-naive cells in ARD than in sarcoidosis patients or controls. 

The expression of IL-7Rα on CD4 T cells implies the proliferation of T cell receptors. Indeed, its lacking and expression of CD25 result in anergic and regulatory CD4 T cells [20]. Since the loss of Treg function and induction of Th17-cells are observed in sarcoidosis, and because the pathogenic conversion of FoxP3 T cells into Th17-cells has been found in ARD, it would be worthwhile to investigate the plasticity of Tregs towards Th17-cells as well as the presence of IL-17-producing Tregs in sarcoidosis.

ARD has been associated with significant reductions in Th-reg levels and the production of autoantibodies [21]. The protective function of Th-reg cells is defective in patients with autoimmune diseases, including vasculitis. As a result, tissue tolerance is broken down, and ongoing immune responses are not reduced in a timely manner [22]. In line with this data, our study first showed decreased Th- and Tc-reg percentages in peripheral blood of IIM, MPA, and GPA but not of sarcoidosis patients or controls. ARD may also be caused by a reduction in circulating Treg that may lower suppressive capacity, affecting the entire immune system and leading to systemic organ manifestations. It is still unclear whether any known autoantibodies are useful for diagnosis and monitoring of disease activity in sarcoidosis, despite the fact that this disease shares many clinical features with other systemic and organ-specific autoimmune diseases.

The innate immune system is activated in sarcoidosis and ARD, leading to T reg cell dysfunction and elevated Th-17 cell count in peripheral blood [23]. Żabińska et al. recently analyzed immune cell profiles of AAV patients, finding a significant increase in Th17 cells and a decrease in regulatory populations [24]. Using these data, our study found that the percentage of Th17 cells in AAV was higher than in IIM and sarcoidosis patients and controls. Immune fingerprints may reflect peripheral tolerance failure, which contributes to neutrophil recruitment and activation associated with the development and progression of AAV. The interleukin-17 (IL-17)-producing Th (Th17) subset is classified as an inflammatory subset of the Th cells, resulting in chronic inflammation of tissues and organ failure as a result. The use of biologics that target the effector cytokines of Th17 cells has been approved to treat certain immune-mediated diseases, which points to the expansion of diseases that are treatable with them. In order to develop novel immunotherapy for Th17-associated inflammatory diseases, it is essential to understand how Th17 cells differentiate and perform their pathogenic functions [25]. 

Despite the value of this new data on the immunological pathways involved in sarcoidosis and B-mediated rheumatic diseases, our study has several limitations: (1) the IIM cohort was small and heterogeneous; (2) our findings are in need of validation for other autoimmune rheumatic disorders by multicentric prospective studies such as rheumatoid arthritis in order to consider in differential diagnosis; (3) the study population was too small to detect any correlations between immunological features and disease activity or severity; (4) there was a lack of lymphocyte subset total counts.

## 5. Conclusions

The etiology and pathogenesis of sarcoidosis remain largely uncharacterized despite extensive research. Autoantigen-specific T cells and autoantibody-producing B lymphocytes may be a sign of autoimmune inflammatory reactions in sarcoidosis due to a number of triggers that contribute to the development of the disease. Our immune cell profiles shed light for the first time on the similarities and differences between sarcoidosis and B-mediated rheumatic diseases. The observation of similar cellular immune dysregulation patterns in sarcoidosis and autoimmune diseases suggests common pathogenetic pathways that may offer an opportunity for further study of autoimmunity and the development of biological therapies to treat sarcoidosis [26].

## Data Availability

The raw data supporting the conclusions of this article is available from the authors on request.

## Supplementary Materials

The following supporting information can be downloaded at: https://www.mdpi.com/article/10.3390/biomedicines11061532/s1, File S1: The gating strategy for T- and B-cell subsets performed through Kaluza Software 2.1.

## Abbreviations

CD-, cluster of differentiation; ARD, autoimmune rheumatic diseases; IIM, idiopathic inflammatory myopathy; AAV, ANCA-associated vasculitis; GPA, granulomatosis with polyangiitis; MPA, microscopic polyangiitis; Th, T helper cells; Treg, regulatory T cells; Tfh-reg, follicular T helper-regulatory cells; BAL, bronchoalveolar lavage.
