# Supplementary material for: Immunological Pathways in Sarcoidosis and Autoimmune Rheumatic Disorders—Similarities and Differences in an Italian Prospective Real-Life Preliminary Study
Source: Biomedicines. 2023 May 25;11(6):1532. doi: 10.3390/biomedicines11061532 (PMC10294995; doi:10.3390/biomedicines11061532)
Supplement: Supplementary file 1 [file biomedicines-11-01532-s001.zip › biomedicines-2413561-supplementary.pdf]

biomedicines

Supplementary Materials

File S1: The gating strategy for T- and B-cell subsets performed through Kaluza Software 2.1

Gate strategy from Kaluza software for B cell panel. Lymphocytes were distinguished based on forward (FSC) versus side (SSC) scatters and additional gating was applied using CD3 versus CD19 to distinguish lymphocytes from cell debris. Three different subtypes of $CD19^{+}$ Bregs were divided based on $CD24^{high}$ $CD38^{high}$ (transitional B cells), $CD24^{high}$ $CD27^{high}$ (B 10 or Breg cells) and $CD5^{+}CD1d^{+}$.

Gate strategy for follicular T cells, effector and central cells from Kaluza software. Lymphocytes were distinguished based on forward (FSC) versus side (SSC) scatters and additional gating was applied to identify $CD3^{+}$. A dot-plot was also assessed to identify $CD4^{high}CXCR5^{high}$ (Tfh cells) and a secondary dot-plot distinguished Tfh1, 2, 17 and 17.1 according to CXCR3 and CCR6. We refer to double negative cells ($CXCR3^{-}CCR6^{-}$) as Tfh2, double positive cells ($CXCR3^{+}CCR6^{+}$) Tfh17.1, $CXCR^{+}CCR6^{-}$ as Tfh1 and $CXCR^{-}CCR6^{+}$ as Tfh17 cells. Th1, Th17 and Th1-like Th17 (Th17.1) cells were defined based on the markers CD45 RA and CCR6, with and without expression of CXCR3 and CCR4. For analysis, total $CD4^{+}$ $CD45RA^{-}$ were divided according to expression of CCR6. $CCR6^{+}$ cells were classified as $CXCR3^{-}CCR4^{+}$ (Th17) and $CXCR3^{+}CCR4^{-}$ (Th17.1), whereas $CCR6^{-}$ cells were classified as $CXCR3^{-}$ $CCR4^{+}$ (Th1) and $CXCR3^{+}$ $CCR4^{-}$ (Th2). Further dot plot was assessed to identify $CD8^{high}CXCR5^{high}$ (Tfc), and a secondary dot-plot distinguished Tfc17, Tfc17.1, Tfc2, Tfc1 according to CXCR3 and CCR4. Referring to double positive cells ($CXCR3^{+}$ $CCR4^{+}$) as Tfc17.1, double negative ($CXCR3^{-}CCR4^{-}$)$^{-}$ as Tfc2, CXCR3 $CCR4^{+}$ as Tfc17, and $CXCR4^{+}$ $CCR4^{-}$ as Tcf1. Total $CD8^{+}$ $CD45RA^{-}$ were divided according to expression of CCR6. Subsequently the $CCR6^{+}$ cells were classified as $CD45RA^{-}$ $CCR6^{+}$ (Tc17.1) and $CD45RA^{+}$ $CCR6^{-}$(Tc17). The total $CD8^{-}$ $CD45RA^{-}$ were classified as $CCR4^{-}CXCR3^{+}$ (Tc2) and $CCR4^{+}CXCR3^{-}$ (Tc1).

Gate strategy for Regulatory T cells panel from Kaluza software. Lymphocytes were distinguished based on forward (FSC) versus side (SSC) scatters and additional gating was applied using SSC versus CD3. A secondary dot-plot was subsequently assessed to distinguish $CD4^{+}$ and $CD8^{+}$ cells. On $CD4^{+}$ cells, four dot-plots were assessed to identify $CD25^{bright}$ $CD127^{-/low}$ (Treg), $CD25^{high}CD127^{high}$ (Th effector), $CD25^{-}CD127^{high}$ (Th naïve) and $CD4^{high}CD25^{high}CXCR5^{high}$ (Tfhreg). On $CD8^{+}$ cells, four dot-plots were assessed to identify $CD25^{bright}$ $CD127^{-/low}$ (Tcreg), $CD25^{high}CD127^{high}$ (Tc effector), $CD25^{-}CD127^{high}$ (Tc naïve) and $CD4^{high}CD25^{high}CXCR5^{high}$ (Tfcreg).
